# β Phase Optimization of Solvent Cast PVDF as a Function of the Processing Method and Additive Content

**DOI:** 10.1021/acsomega.4c01221

**Published:** 2024-06-03

**Authors:** Miray Yasar, Patrick Hassett, Neal Murphy, Alojz Ivankovic

**Affiliations:** † School of Mechanical and Materials Engineering, University College Dublin, Dublin, Ireland

## Abstract

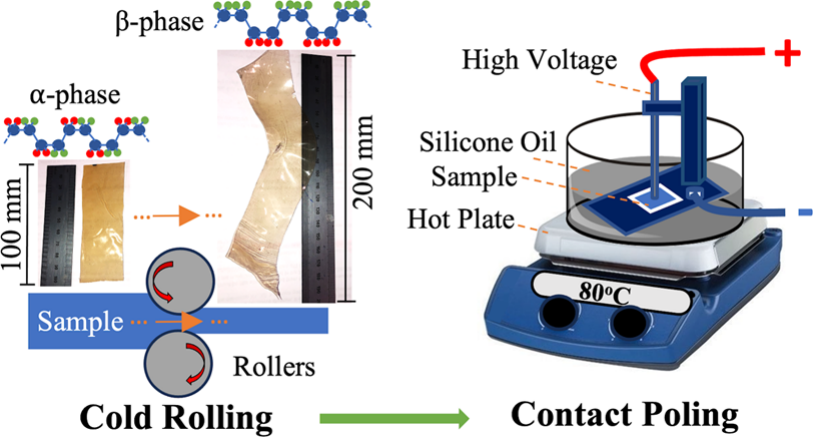

A semicrystalline polymer with high piezo-, pyro-, and ferroelectric characteristics, poly(vinylidene fluoride) (PVDF) offers exciting possibilities in various applications. The semicrystalline structure of PVDF is composed of several phases including α, β, θ, γ, and ε phases. β phase polymorphs of PVDF exhibit the highest piezoelectric properties, which can be enhanced through different processing methods. This study aims to investigate the β phase transformation of PVDF through different processes/treatment methods and the processing of a PVDF polymer composite containing 0.2 wt % multiwalled carbon nanotubes and/or 20 wt % modified/unmodified barium titanate. The effects of annealing, uniaxial stretching, rolling, atmospheric plasma treatment, UV treatment, and their combinations were investigated. The transformation of α to β phase was determined by Fourier transform infrared spectrometer, X-ray diffractometer and differential scanning calorimeter. The most remarkable β phase transformation of PVDF films was obtained by stretching following solvent casting and hot pressing. It was observed that various process combinations, as well as the incorporation of additives, influence the β phase content of PVDF. Alongside studying β phase content of PVDF, the investigation extends to analyzing the tan δ and elastic and loss modulus values of rolled PVDF polymer composite films.

## 1 Introduction

Piezoelectric materials are capable of directly transducing electrical and mechanical energy.^1^ The piezoelectric effect is based on the intrinsic properties of the unit cell or atomic structure of a material. This is the smallest unit of a material that combines to form the macroscopic system. The intramolecular forces of a piezoelectric material must be polar, with positive and negative charge centers in such a way that they effectively cancel each other out, yielding a net nonpolar structure. Furthermore, the material must exhibit an absence of inversion symmetry. These two requirements combined provide the conditions necessary for the piezoelectric effect. When the unit cell is deformed, the centers of positive and negative charge shift in opposite directions, generating an electric field.^2^ Piezoelectric single crystals and ceramics are commonly used for actuating and sensing purposes due to their outstanding performance. However, they are typically brittle and have poor flexibility for shaping. To overcome these problems, piezoelectric polymer nanocomposites have been developed through the incorporation of ceramic fillers such as barium strontium titanate ceramic powder,^3^ lead magnesium niobate-lead titanate solid solution (PMN–PT), lead zinc niobate-lead titanate (PZN–PT), lead zirconate titanate (PZT),^4^ sodium potassium niobate (KNN), zinc oxide (ZnO),^5^ and bismuth sodium titanate (BNT) into the polymer matrix.^6^ Additionally, multiwalled carbon nanotubes (MWCNTs),^7−11^ graphene oxide,^12,13^ polyaniline (PANI),^10,13,14^ and polyacrylonitrile (PAN)^7,8^ have also been used in the production of piezoelectric polymer nanocomposites to enhance the piezoelectric properties of the material. Zhu et al. showed that with increasing wt % of MWCNTs, enhanced β phase content and crystallinity were promoted.^15^

Piezoelectric polymers such as poly(vinylidene fluoride) (PVDF), polyvinylidene-trifluoroethylene (PVDF-TrFE), poly(vinylidene fluoride-hexafluoropropylene) (PVDF-HFP), poly(vinylidene cyanide) (PVDCN), poly(vinylidene cyanide-vinylacetate) (PVDCN-Vac), polyamides (PA), poly(lactic acid) (PLA), cellulose, and derivatives are often preferred because of their flexibility to bending and twisting under high strain in addition to their lightweight, low density, low cost, and low refractive index characteristics.^6^ PVDF is very flexible, exhibits good stability over time, and does not depolarize when subject to high alternating electric fields.^2^ PVDF β-phase is an all trans-planar zigzag conformation (TTTT) of fluorine atoms, while PVDF α phase is a trans–gauche twist conformation (TGTG′). The distance between two fluorine atoms for this phase is twice as large as the van der Waals radius of fluorine atoms. However, in the β-phase, the distance between two fluorine atoms equals the fiber period of repeating units (2.56 Å). This means the fluorine atoms in the β phase tend to move away from each other due to the repulsion of like charges. As a result, forming the β phase is more complex than the α phase conformation due to the lower intramolecular energy of the β phase.^16^

In the last few decades, several different processing methods and their combinations have been studied to achieve a higher β phase content of PVDF, such as solvent casting,^17−26^ stretching,^16,18−20,22,24,27,28^ poling,^17,21,29^ quenching,^19,30−32^ compression,^16,20,22,23,31,33^ roll hot pressing,^34^ rolling,^29^ melting,^17,19,25,27^ the application of magnetic fields,^26^ annealing,^23,29,31^ folding,^31^ and spin coating.^30,32,35^ The β phase exhibits an all-trans conformation of the polymer chain, resulting in a polar, noncentrosymmetric structure that permits piezoelectricity. Thus, increasing β phase content of PVDF is crucial for piezoelectric applications.^1^

Ultraviolet (UV) treatment^36^ is a photonic annealing technique that can enhance the β phase content of PVDF. In this study, the effect of atmospheric plasma treatment is also investigated, which is assumed to operate as a rapid annealing step similar to UV treatment. Rapid annealing techniques result in energy transfer, increasing the β phase content of PVDF.^36^

This study aims to enhance the β phase content of PVDF through a combination of diverse processing methods, including solvent casting, hot pressing, annealing, UV treatment, plasma treatment, quenching, stretching, rolling, and PVDF polymer composite production. It explores the structural, thermal, and mechanical characteristics of the resulting materials. To the best of our knowledge, prior research has not delved into the impact of plasma and UV treatment on PVDF that has undergone prior processing via solvent casting, hot pressing, and drawing. Furthermore, no comparative analysis has been conducted to assess the performance of PVDF polymer composite films processed through the optimal method from the aforementioned techniques. The study also scrutinizes the influence of each processing step on β phase content and % crystallinity degree. This work introduces novelty by utilizing varied processing methods and different formulation additives in the preparation of PVDF polymer composite films, investigating their effects on β phase content, crystallinity, tan δ, as well as elastic and loss modulus values.

## 2 Experimental Section

### 2.1 Materials

Poly(vinylidene fluoride) (PVDF) (*M*_w_ = 64.03 g/mol) and BaTiO_3_ (BT) were purchased from abcr Gute Chemie. Multiwalled carbon nanotubes (MWCNTs) were supplied by Graphene Supermarket. *N*,*N*-Dimethylformamide (DMF) was used as a solvent and purchased from Sigma-Aldrich. BT particles were modified using a (3-glycidyloxypropyl)trimethoxysilane (GPTMS) coupling agent provided by TCI Chemicals. Methanol (CH_3_OH) and acetic acid were also used during the modification of BT particles, supplied by Thermo Fisher Scientific and MB Chem Corporation, respectively. Silver conductive ink was purchased from Thermo Fisher Scientific for use as electrodes.

### 2.2 Preparation of PVDF with Different Processing Methods

Samples were produced by solvent casting. Subsequently, the effects of various post-processing methods were investigated. These methods included hot pressing, quenching, annealing, stretching, rolling, plasma treatment, UV treatment, and combinations of these processes. Process parameters and the preparation steps are detailed in Table 1.

**Table 1:** Processes Performed to Increase the β Phase Content of PVDF

process name	equipment and process parameters
solvent casting (SC)	equipment: mechanical mixer | oven
	solution: 20 wt % PVDF + DMF (2 h at 50–55 °C at 50 rpm)
	drying: ambient temperature for 5 days and 80 °C for 4–6 h
hot pressing (HP)	equipment: Stenhoj hydraulik hot press
	process: 180 °C for 5 min at 350 kN (4 times; *t* ∼ 300 μm)
quenching (Q)	water bath at ambient temperature
annealing (A)	equipment: hot press
	process: 120 °C for 1 h
stretching (S)	equipment: Instron 8501 universal tensile testing machine
	process: 100 mm/min at 80 °C with *L*_final_/*L*_initial_ = 2–3
rolling (R)	equipment: rolling mill with 2 sheet rollers (75 × 41 mm^2^ diameter) 50–55 HRC hardened EN31 grade steel
	process: ambient temperature until reaching final thickness ∼100–110 μm
plasma treatment (PT)	equipment: Janome JR 2400 N atmospheric plasma treatment instrument
	process: both surfaces of the PVDF films were plasma treated
	details are listed in Table S1 in the Supporting Information
UV treatment	equipment: Radionics U.V. exposure unit 555–279 with two tubes which emits 350–400 nm range with a sharply defined peak at 360 nm
	process: 48 h

### 2.3 Silane Functionalization of BT

The BT particles were treated with 1 wt % GPTMS silane coupling agent to see the effect of surface modification on the distribution of BT particles. GPTMS consists of an epoxide functional group in which the C–O bonds are highly polar due to the high electronegativity difference between hydrogen and the oxygen atom.^37^ It was used to investigate the possible reaction between epoxy functional groups and the polar β phase network of PVDF. 100 mL of CH_3_OH/H_2_O (90:10 v/v) was mixed with a silane coupling agent. Acetic acid was used to adjust the pH of the solution to 4.5–5. After hydrolysis for 1 h, BT was added to the solution and ultrasonicated at room temperature for 30 min at 60 Hz. The solution was mixed for 2 h at 60 °C using a mechanical mixer. The CH_3_OH/H_2_O treating solution was then evaporated at 80 °C. This was followed by drying modified BT (mBT) particles in an oven at 110 °C for 24 h.

### 2.4 Preparation of PVDF Polymer Composite Films

A solution of PVDF+DMF was initially prepared as given in Table 1 solvent casting-solution section to prepare polymer composites.

#### 2.4.1 BT/mBT+PVDF and MWCNT+PVDF Polymer Composite

For the preparation of BT/mBT+PVDF composite and MWCNT+PVDF composite, 20 wt % BT or mBT and 0.2 wt % MWCNTs, respectively, were added to the PVDF+DMF solution and mixed at 50–55 °C. A mechanical mixer was used at 200 rpm for 2 h, followed by an additional 30 min of ultrasonic mixing.

#### 2.4.2 MWCNT+BT+PVDF Polymer Composite

A certain amount of MWCNTs was added to the BT/mBT+PVDF solution prepared in section 2.4.1, mixed for a subsequent 2 h at 200 rpm, and ultrasonicated for an extra 30 min.

#### 2.4.3 Polymer Composite Film Production

Samples were dried as given in Table 1 solvent casting-drying section. After drying, PVDF polymer composite films were prepared by the SC-HP-Q-R sequence since composite materials could not be stretched to the same degree as PVDF films and tore during the stretching procedure. Rolling was easy to perform and provided the most favorable chain alignment,^38^ and the thickness of the materials was decreased to 100–110 μm from 300 μm.

### 2.5 Poling Process

The contact poling unit was custom-built in the laboratory to align the dipole moments of stretched/rolled PVDF and rolled polymer composite films. The setup consisted of four parts: an in-house designed poling unit, a high voltage power supply (Branderburg, Alpha III, 30 kV-1.5 mA), a silicone oil bath, and a hot plate.

Before the poling process, PVDF films were cut into samples measuring 30 × 15 mm^2^, and silver conductive ink was applied onto the upper and lower surfaces to act as electrodes. The ink was contained within 2 mm from the edges of the PVDF film to avoid contact between the two electrodes. The silicone unit was heated to 80 °C, and the material was immersed in the silicone oil for 30 min to achieve uniform temperature and chain movement throughout the sample. The samples were placed between a spring-loaded steel rod and the bottom aluminum plate during the poling process, and a high voltage of 100 V/μm was applied for an additional 30 min.

### 2.6 Analyses

FTIR spectra for the films were recorded on a Thermo Fisher Scientific Summit Pro FTIR spectrometer by accumulating 64 sample scans with a resolution of 4 cm^–1^ over a range of 700–1600 cm^–1^. Morphology analysis was conducted using a Hitachi TM4000 Tabletop SEM at an accelerating voltage of 15 kV. XRD analysis was performed using a Siemens D500 X-ray diffractometer from angles of 3–50°, 0.02 step size (degree), and 1 s/step dwell time. Thermal analysis was performed on a Netzch 214 Polyma differential scanning calorimeter (DSC) from −100 to 200 °C with 10 K/min heating and cooling rates. The samples were scanned twice, with the second cooling and heating curves reported. Dynamic mechanical analysis was performed using a DMA 242E Artemis from −100 to 150 °C with a heating rate of 2 °C/min under 1 Hz. The piezoelectric behavior of the stretched and rolled films, including polymer composite films, was measured using the Piezotest PiezoMeter System applying 0.25 N force and 110 Hz frequency onto the film.

## 3 Results and Discussion

### 3.1 FT-IR Analyses and Morphology

**Figure 1:**
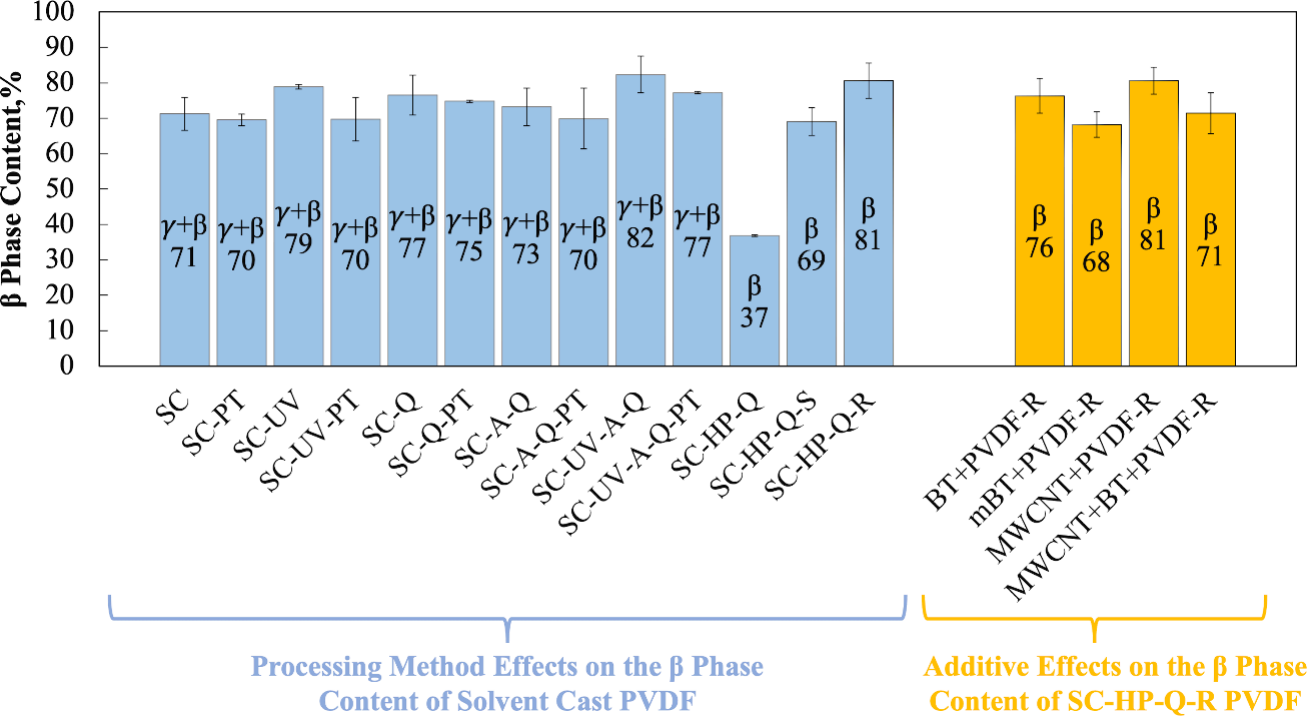
β phase content of PVDF films processed by different methods and PVDF polymer composite films processed by SC-HP-Q-R.

The results of β phase content of PVDF films processed by different methods and rolled PVDF polymer composite films are provided in Figure 1. The Beer–Lambert law given in eq 1(21) was used to calculate the β phase content. The % α and β phase was calculated using absorbance peaks at 766 cm^–1^ for *A*_α_ and 840 cm^–1^ for *A*_β_.
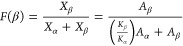
1where *K*_α_ = 6.1 × 10^4^ for α = 766 cm^–1^ and *K*_β_ = 7.7 × 10^4^ for β = 840 cm^–1^.

#### 3.1.1 Processing Method Effect

A high γ+β phase content (71%) was obtained upon solvent casting the material before any processing. The γ+β content was calculated also by using eq 1 due to having a common peak at 840 cm^–1^ for both γ and β phases. Morali et al. used the same equation to calculate γ+β content.^39^ According to Cai et al., an additional characteristic peak at 1233 cm^–1^ has to be monitored to distinguish the γ phase.^40^ Thus, β phase or γ+β phase can be quantified.

Plasma and UV treatments conducted thermal transitions. Following thermal treatment, an increase in the γ+β phase content of PVDF was expected. However, plasma treated samples exhibited a 3–6% decrease or no significant change in γ+β phase content for solvent cast materials. UV treated samples showed a 3–9% increase in β phase content. UV treatment acted as a photonic annealing step, promoting the phase transformation of PVDF films from the non-ferroelectric α phase to the ferroelectric β phase.^36^

Annealing after UV treatment was demonstrated to process high γ+β phase content films (82%). This might suggest that annealing may be a viable method of processing the solvent cast films into thin, flexible materials. After annealing and hot pressing, quenching was necessary to maintain the β phase content during cooling. If the material was allowed to cool slowly, re-emergence of the α phase would occur, and the high porosity of the material would be restored.

Hot pressing resulted in the formation of very low β phase content (37%) for solvent cast samples. Methodologies must be investigated to restore the β phase. Rolling and stretching recovered the β phase content while reducing the α phase. After processing the samples by SC-HP-Q, stretching the samples resulted in a dramatic increase in β phase content from 37% to 69%. Likewise, the β phase content of the same samples increased to 81% following rolling. Yang et al. obtained 84.7% β phase content by cold rolling, which is in agreement with our result.^41^

FTIR results of different process methods are given in Figure S1 in the Supporting Information. These results are supported by XRD analyses, which will be discussed in the following section.

Figure S1 shows the FTIR analysis of materials processed by solvent casting, followed by different methods. The peaks at 761 cm^–1^ (CF_2_ bending and skeletal bending) and 795 cm^–1^ (CH_2_ rocking) were assigned to the α phase, whereas that at 840 cm^–1^ was significantly larger as it represents the electroactive β and/or γ phases.^40^ Hence, the other characteristic peaks for β and γ phases were used to distinguish the phases in the structure. The peak at 1276 cm^–1^ was characteristic of the β phase. Peaks at 1233 and 833 cm^–1^ exclusively represented the γ phase, and the prominent peak at 873 cm^–1^ represented the combination of all three phases.^42^ Since the solvent cast samples showed a peak at 1233 cm^–1^, these include both γ+β phases.

Plasma treated samples exhibited clear peaks at 761 and 795 cm^–1^, which are characteristic of the α phase. These peaks were not explicitly present in the spectra of the samples which were not subjected to plasma treatment.

**Figure 2:**
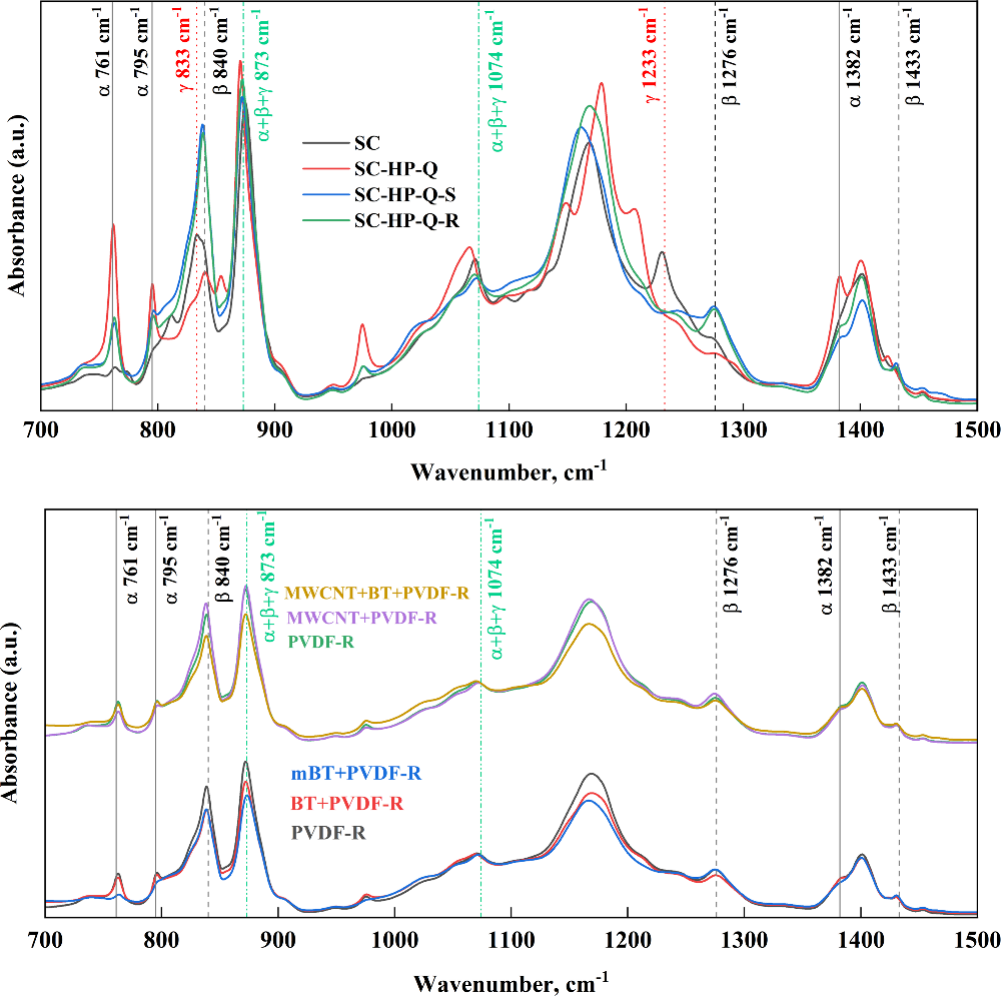
FTIR analyses of (a) PVDF films processed by SC, SC-HP-Q, SC-HP-Q-S, and SC-HP-Q-R. (b) PVDF polymer composite films processed by SC-HP-Q-R.

Figure 2a represents the results of FTIR analysis for samples processed by SC, SC-HP-Q, SC-HP-Q-S, and SC-HP-Q-R. Solvent cast materials exhibited high α and β phase peaks. A sharp α phase related peak at 761 cm^–1^ was obtained following hot pressing that was not present in the FTIR spectra of the unprocessed solvent cast samples, indicating a loss of the majority of the β phase content following hot pressing. Additionally, solvent cast PVDF films showed a γ phase peak at 1233 cm^–1^, which indicates the combination of dominant γ+β phases. However, after hot pressing, this peak was not present. SC-HP-Q-S and SC-HP-Q-R samples displayed a sharp peak at 840 cm^–1^, characteristic of the β phase, which was confirmed by the 1276 cm^–1^ characteristic β phase peak. The α phase of these samples was less than that in the SC-HP-Q samples. Furthermore, the γ phase (833 cm^–1^) was absent from hot pressed samples, but the α and β phases were present.

**Figure 3:**
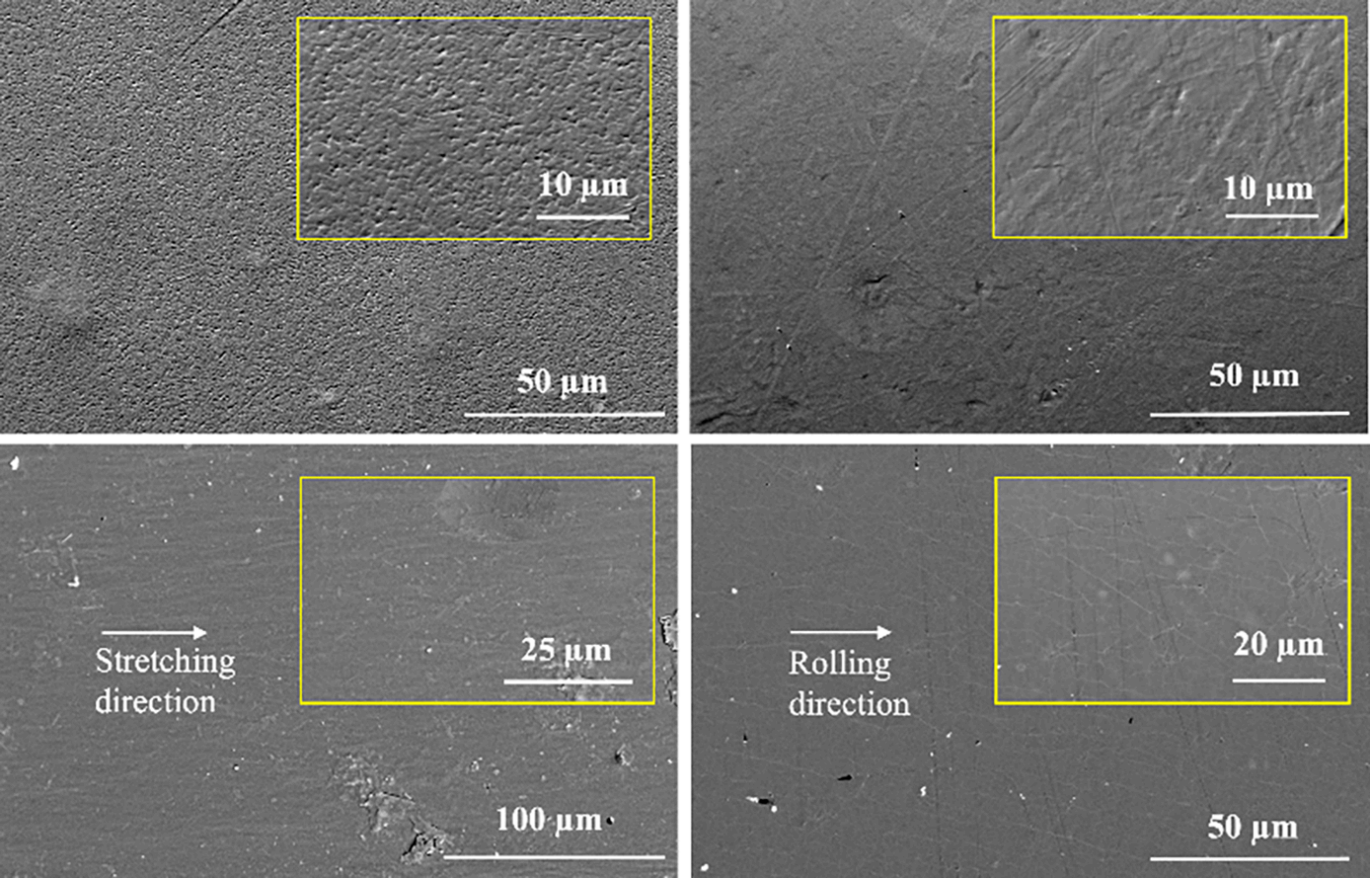
SEM micrographs of PVDF films processed by (a) SC, (b) SC-HP-Q, (c) SC-HP-Q-S, and (d) SC-HP-Q-R.

Figure 3 shows the SEM images of the morphology of PVDF films processed by SC, SC-HP-Q, SC-HP-Q-S, and SC-HP-Q-R, which compares the phase transitions of PVDF with different processing methods. It was observed that solvent cast PVDF exhibited a porous structure (Figure 3a) due to the evaporation of DMF solvent. The morphology shows the dominant γ+β phases within the structure, which were also verified with FTIR results. The pores and globular microstructures were diminished, and a more uniform surface was obtained after hot pressing (Figure 3b, SC-HP-Q)). High temperature and pressure resulted in the formation of a more uniform microstructure by remelting present defects.^22^

After stretching and rolling the SC-HP-Q PVDF samples, the clear spherulitic arrangement of PVDF could no longer be distinguished and the samples transformed to a β phase microfibrillar morphology, represented in Figure 3c,d, respectively. The spherulitic microstructure of PVDF was destroyed, and an oriented morphology was developed along the stretching directions. The results are in agreement with Mishra et al.’s study.^22^ However, as Yang et al. explained in their study, rolled samples exhibited smaller crystallite sizes, consequently affecting the piezoelectric properties.^41^ Surface cracks and some deformations were observed along the rolling direction.

#### 3.1.2 Additive Effect

PVDF polymer composites containing MWCNTs and BT/mBT underwent a rolling process following the SC-HP-Q sequence. The decision to roll rather than stretch the samples was influenced by the tendency of the materials to tear before reaching the desired *L*_final_/*L*_initial_ = 2–3 during stretching.

The effects of filler addition are illustrated in Figure 2b. PVDF-R represents the samples processed with the SC-HP-R sequence. The α phase peaks at 761, 795, and 873 cm^–1^ decreased upon the addition of BT/mBT. The β phase characteristic peak at 840 cm^–1^ was also observed to decrease. However, the β phase distinct peak at 1276 cm^–1^ increased slightly. Adding MWCNTs increased the β phase related peaks to a greater extent than adding BT by acting as a nucleating agent for the β phase, and adding mBT resulted a decrease in the β phase upon rolling.

Upon adding BT, mBT, or MWCNTs and BT together, β phase contents in the samples were obtained similar to the PVDF-only sample (PVDF-R) that was solvent cast, hot pressed, and rolled. However, the addition of MWCNTs increased the β phase content of the material by 5% compared to the PVDF-R samples, significantly facilitating the rolling of the material.

**Figure 4:**
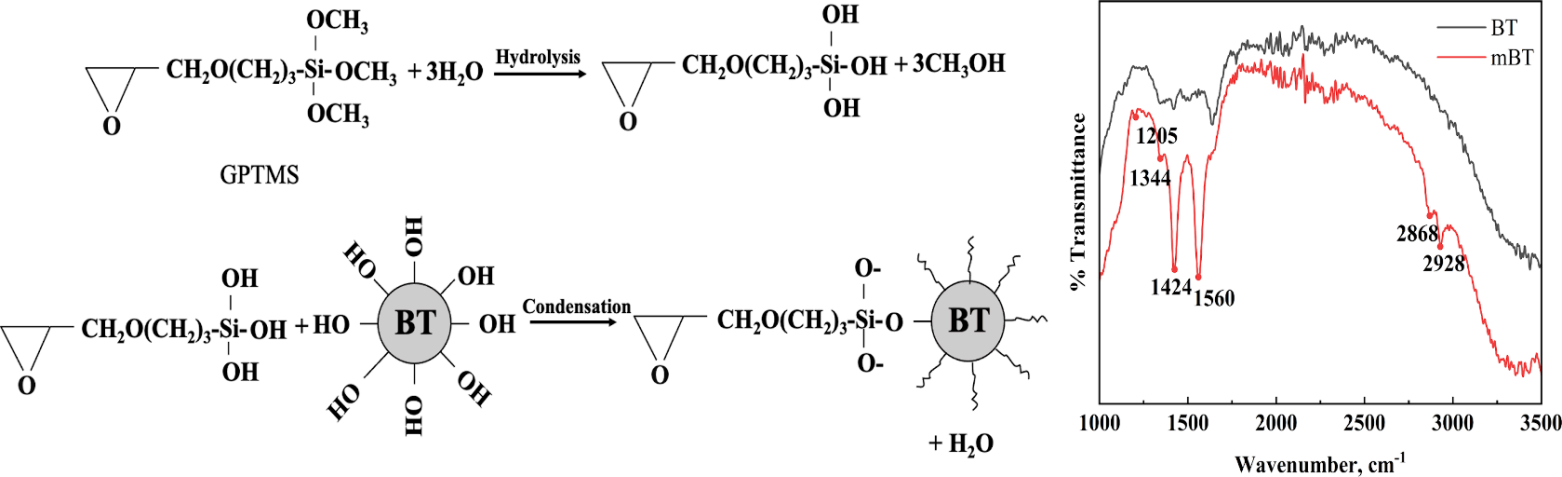
Schematic diagram of (a) hydrolysis of silane coupling agent, (3-glycidyloxypropyl)trimethoxysilane (GPTMS), and (b) condensation reaction of BT and GPTMS. (c) FTIR spectrum of BT and mBT.

Modification of BT (Figure 4a,b) was observed to decrease the β phase content of PVDF by 8%. This might be because the presence of GPTMS on the surface of BT particles created barriers between the mBT and PVDF matrix instead of creating a bridge-like structure between the polymer and BT. Thus, efficient stress transfer and polarization alignment for enhanced piezoelectric properties were hindered. Another possible reason might be that the surface modification of BT altered the crystal structure and disrupted the formation of the β phase in PVDF.

Silanization of BT was characterized using FTIR. A schematic representation is provided in Figure 4c. Peaks at 1344 cm^–1^ (C–H), 1424 cm^–1^ (CH_2_–bending mode), and 1560 cm^–1^ (C–C) represented stretching in the epoxide ring. The 2868 cm^–1^ peak was symmetric stretching of CH_2_, while the 2928 cm^–1^ one was symmetric stretching of CH_3_. The characteristic peaks due to Si–O–Si stretch vibration were observed at a 1205 cm^–1^ wavenumber.^43^

### 3.2 XRD Analyses

XRD patterns were examined to determine the crystalline phases in the films that arose through different processing methods.

#### 3.2.1 Processing Method Effect

The XRD results of solvent cast PVDF processed using different methods are given in Figure S2 in the Supporting Information. Peaks were observed at 18.4 (020) and 19.8 (110), corresponding to the α phase, 20.3 (101) and 19.1 (002), corresponding to the γ phase, and 20.8 (110), corresponding to the β phase of PVDF.^44^ A peak observed at 30 degrees is a reflection of the β phase at the (201) crystal plane.

Plasma treatment led to a decrease in all phases provided that quenching was not carried out beforehand. This indicates that quenching was a crucial step in preserving the phases (a result supported by FTIR analysis). UV treatment resulted in an increase in the β phase reflection at 30 degrees. This increase was observed in all samples subject to UV treatment. The increase in β phase was also supported by FTIR analysis. Stretching of the films was observed to increase the intensity of the β phase peaks. The sample that displayed the highest 30-degree reflection related to the β phase content was processed by the UV-A-Q process.

**Figure 5:**
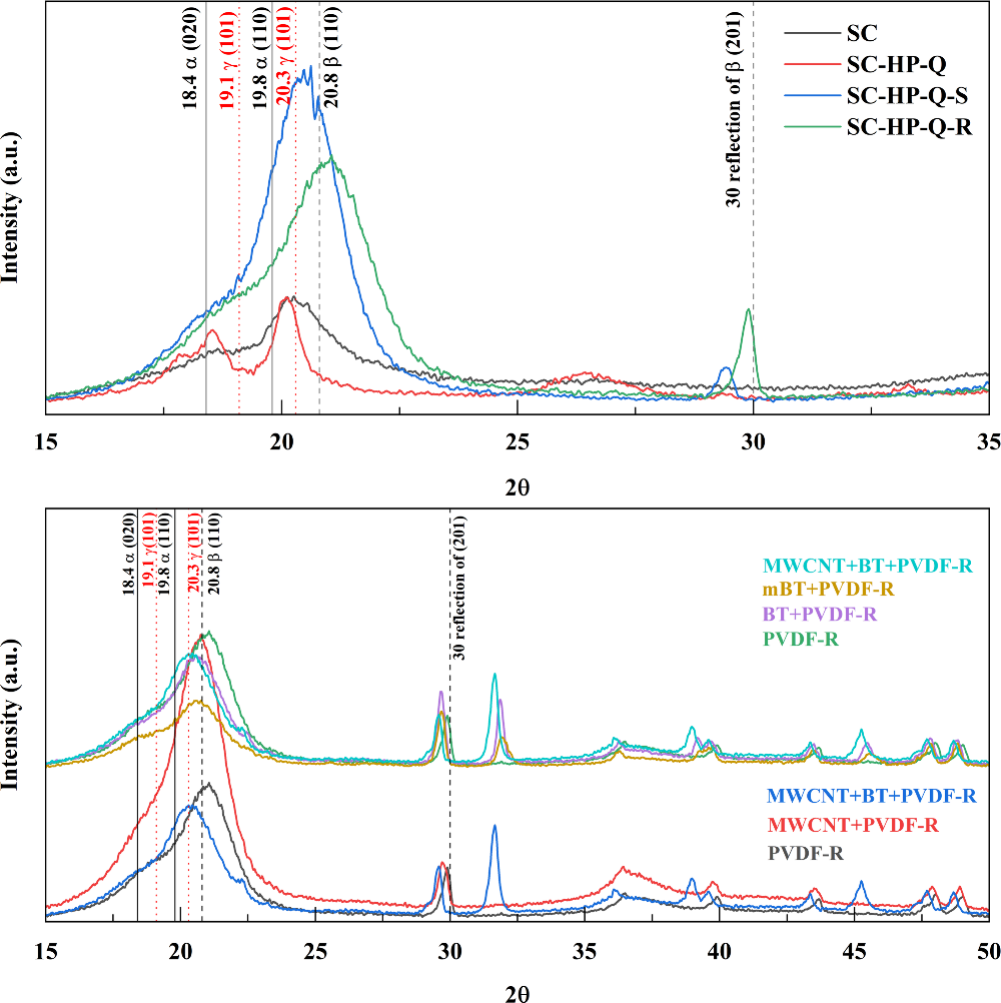
XRD analyses of (a) PVDF films processed by SC, SC-HP-Q, SC-HP-Q-S, and SC-HP-Q-R. (b) PVDF polymer composite films processed by SC-HP-Q-R.

The effect of hot pressing, stretching, and rolling on the phase content of the films can be deduced from Figure 5a. Before hot pressing, the solvent cast film exhibited its most prominent peak at 20.3 degrees (γ phase). After hot pressing, a peak appeared at 18.4 degrees related to the α phase. This indicated a conversion of γ–β to α phase during the hot pressing process in agreement with the FTIR results. When the film was stretched, the α phase characteristic peak disappeared, and the γ and β phases exhibited a significant increase, marking the α to β phase transformation as the sample necks. It demonstrated that stretching was a viable method of increasing the β phase content of PVDF. Rolling was observed to alter the crystalline structure of PVDF. While solvent cast and stretched materials exhibited a γ phase-related peak at 20.3 degrees, a β phase peak at 20.7 degrees was observed in rolled materials. This result indicated that the mechanical rolling process partially deteriorated the primary crystal structure but induced a longitudinal deformation of the polymer chains in the crystals.^41^

#### 3.2.2 Additive Effect

The effects of filler addition were considered in Figure 5b. The addition of MWCNTs was observed to increase the β phase content. However, the samples containing BT did not exhibit the same improvement in the β phase following rolling. An extra peak at 31.92 (110/200), characteristic of the polar β phase, was observed for rolled samples containing BT.^45^ Additionally, the 30-degree β phase reflection was observed, and the results of FTIR previously suggested that materials containing BT also possess a high β phase content following rolling. The surface modification of BT induced alterations in the overall crystalline structure, consequently impeding the formation of the β phase within the PVDF matrix.

### 3.3 DSC and DMA Analyses

**Figure 6:**
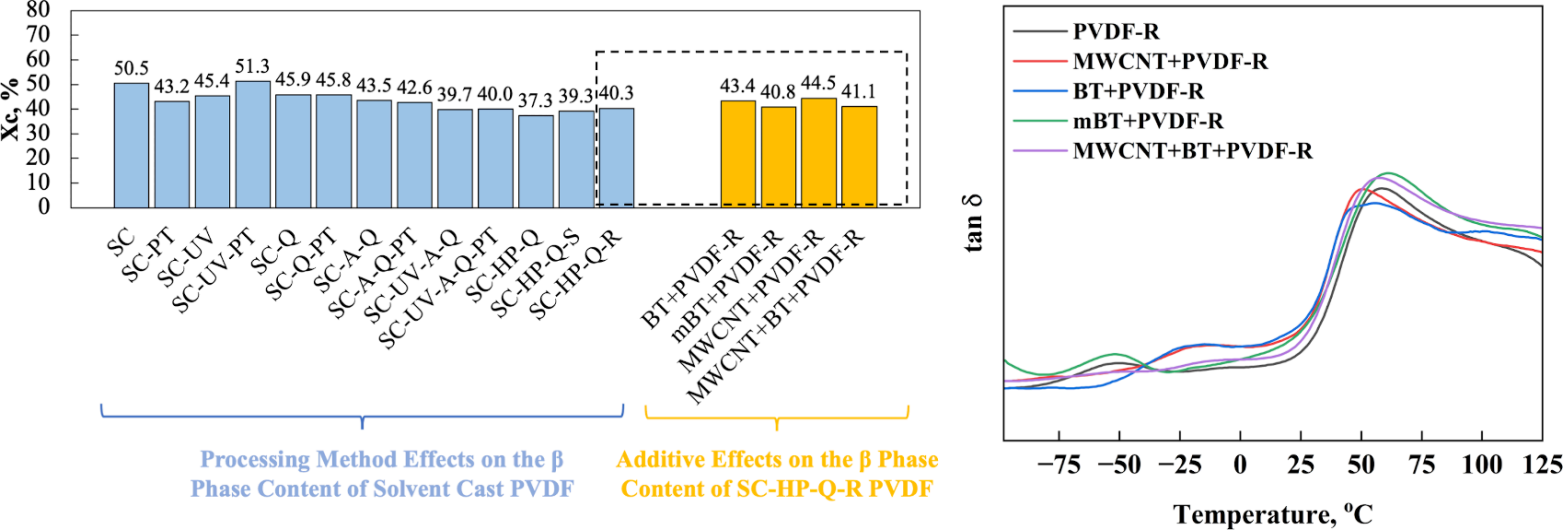
(a) % Crystallinity values of PVDF films processed with different processing methods and (b) tan δ values of PVDF composite films processed by SC-HP-Q-R.

The degree of crystallization (*X*_c_) of PVDF and PVDF polymer composite films was calculated using eq 2.^10^
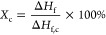
2where Δ*H*_f_ is the heat of fusion of the sample and Δ*H*_f,c_ (i.e., 104.7 J/g) is the heat of fusion of 100% crystalline PVDF. The crystallinity and melting temperatures of the samples are provided in Table S2 in the Supporting Information. A schematic representation of *X*_c_ is provided in Figure 6a.

#### 3.3.1 Processing Method Effect

The highest degree of crystallinity was obtained from samples subjected to SC-UV-PT. In general, plasma treatment increased the heat of fusion and the degree of crystallinity due to an increase in both α and β phases. Annealing was not observed to result in a significant change in crystallinity. Hot pressing decreased the material’s crystallinity, indicating the recrystallization and phase transformation occurring during the process. It was observed that the degree of crystallinity slightly decreased with stretching and rolling due to melting in hot press and posterior recrystallization along the draw/roll direction of the polymer chains.^18^

#### 3.3.2 Additive Effect

MWCNTs acted as a nucleating agent for crystallization. Hence, the total crystallinity increased from 40.3% to 44.5% upon adding MWCNTs to the PVDF, as the number of nuclei for crystallization was higher.^46^ As Zhu et al. explained in their study, MWCNTs addition increased crystallinity but maintained the β phase content almost stable, which indicates that MWCNTs promoted both α and β phases while affecting crystallinity.^15^ BT particles also acted as nucleating sites for the β crystalline phase. As BT particles possessed positive surface charge, they tended to absorb negative dipoles; hence, β-phase crystals were observed to form as these have the highest dipole moment of all the phases. These crystals tended to grow and dominate on the surface until the addition of a certain concentration of BT.^47^ The addition of 20 wt % BT was observed to increase the crystallinity of the material slightly. However, modifying BT particles created a coupling agent barrier through the polymer matrix, resulting in a decrease in the enthalpy of fusion and crystallinity over the sample containing unmodified BT. Upon rolling the mBT+PVDF material, the degree of crystallinity and heat of fusion was observed to decrease in the samples processed by hot pressing after solvent casting, indicative of the partial deterioration of the crystalline structure during the procedure.^22^

Although the addition of MWCNTs alone resulted in a significant increase in crystallinity, the negative effect of BT addition on crystallinity was more dominant than MWCNTs in MWCNT+BT+PVDF polymer composite films, resulting in an overall reduction in crystallinity for this sample.

The tan δ results for PVDF polymer composite films are shown in Figure 6b. A relaxation process with a maximum tan δ was detected at −51.56 °C for PVDF-R samples. This relaxation resulted from the cooperative segmental motions within the main chains of the amorphous regions.^48−51^ A second relaxation (α_c_) was observed above 50 °C, peaking at 57.4 °C for the PVDF-R samples associated with motion within the crystalline fraction of the sample. The α_c_ relaxation was accompanied by diffusion processes involving chains in the amorphous region, and a delayed deformation response of material was observed as a result of the second relaxation. This second relaxation was not observed as a definitive peak in the plot of tan δ as a result of the morphology of the crystalline fraction that was highly oriented. The height of the tan δ peak relates to the fraction of the amorphous phase and the architecture of the crystal phase.^48−51^

The melting temperatures of SC and SC-HP films were higher than those that were subsequently stretched (Table S2). Higher melting temperatures typically indicate the presence of the β phase.^22^ However, such an analysis alone is insufficient to provide a complete qualitative description of the phase content of the material, as melting temperature is also dependent upon the crystallization history.^22^ Furthermore, regioisomeric defects in the material influence the melting peak of the α phase more than the β phase, shifting the characteristic peak of the α phase to the β phase peak in highly disordered crystal structures.^22^ Therefore, it could be assumed that lower melting temperatures represent a higher β phase content with higher piezoelectric response for mechanically stretched films. Such findings agreed with the results obtained by FTIR and XRD; the β phase content increased incrementally following stretching/rolling and poling. It should be recognized that DSC is not typically used to differentiate between α and β phases but to quantify the crystallinity of the material.^52^

**Figure 7:**
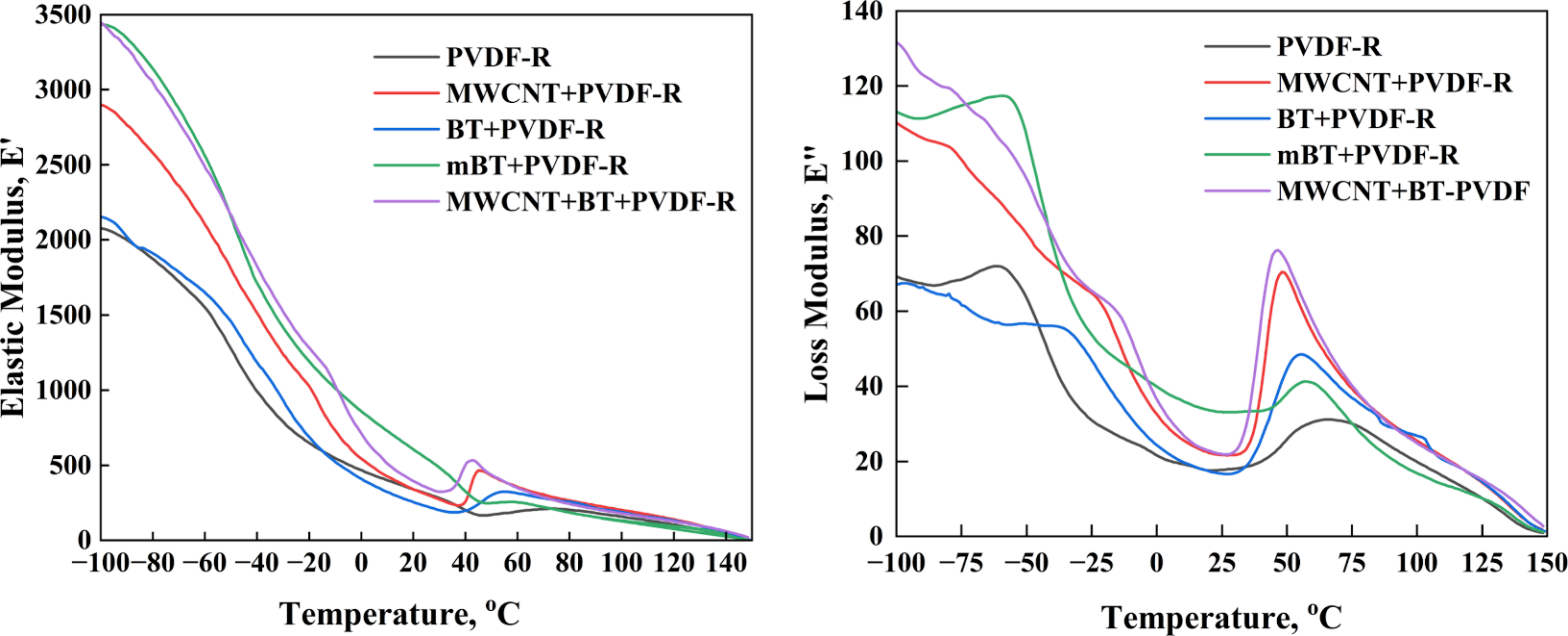
Elastic (a) and loss modulus (b) of PVDF composite films processed by SC-HP-Q-R.

Adding MWCNTs increased the average value of the elastic modulus, *E*′ (Figure 7a). This was unsurprising, as it would be expected that the *E*′ of PVDF would be improved by the dispersion of MWCNTs in the polymer matrix as MWCNTs possess a high aspect ratio.^53^

The loss modulus, *E*″, of the polymer was also expected to increase upon the addition of particles. The internal friction between the particles and the matrix interface during the application of periodic stress was dissipated in the form of energy and increased the value of *E*″.^54^ The lowest average loss modulus was observed in the samples containing BT, similar to the PVDF-R sample (Figure 7b). The reason for this could be possible agglomerations in the matrix. When the modification was applied to the BT particles, the loss modulus decreased because the coupling agent did not aid in distributing the BT particles.

### 3.4 *d*_33_ Analyses

*d*_33_ analyses were performed for rolled or stretched materials. The results of *d*_33_ measurement are given in Table 2. Electrical arcing was observed upon applying an electric field to solvent cast materials, preventing the desired dipole alignment. The possible reason was that the tolerance of porous PVDF to strong electric fields was low, and the material was easily destroyed by arcing and flashover.^55^

**Table 2:** *d*_33_ of PVDF Films Processed by Different Methods and Polymer Composite Films Processed by SC-HP-Q-R

process	*d*_33_, pC/N
PVDF (SC-HP-Q-S)	9.2 ± 2.3
PVDF (SC-HP-Q-R)	3.3 ± 2.0
BT+PVDF-R	3.9 ± 1.9
mBT+PVDF-R	2.8 ± 0.9
MWCNT+PVDF-R	4.7 ± 1.8
MWCNT+BT+PVDF-R	3.5 ± 1.4

After stretching or rolling the SC-HP-Q material, poling was successfully performed, providing a material exhibiting piezoelectric behavior with a 9.2 pC/N *d*_33_ piezoelectric coefficient for SC-HP-Q-S films. Prior to the poling process, the original films had a zero piezoelectric constant. Thus, poling was essential to obtain a piezoelectric material. However, poling could not be applied to the samples processed by the other methods due to random orientation, highlighting the importance of stretching and rolling operations to obtain polarizable materials. The rolled and stretched specimens had laminar orientations in which the crystallites tended to rotate toward the direction of the electric field. Thus, the piezoelectric activity of the stretched and poled or rolled and poled films improved.^41^ Tao et al. stretched a PVDF film after producing the film by 3D printing. They obtained a piezoelectric coefficient of 7.29 pC/N with 65% β phase content, which is 10–100 times higher than the related reported values.^28^

Interestingly, while stretched materials exhibited a higher piezoelectric coefficient compared to rolled materials, it is noteworthy that stretched films possessed lower β phase content than rolled films. This may be due to obtaining smaller crystallite sizes or cracks in the chain at the surface of the crystallite,^41^ as shown in SEM micrographs. Yang et al. also obtained a higher *d*_33_ value after rolling the samples, which was 8 pC/N for 84.7% β phase content with the cold rolling method.^41^

Among composite films, the highest piezoelectric coefficient was obtained by MWCNT+PVDF-R composites with 4.7 pC/N. MWCNTs addition into PVDF and rolling the film increased *d*_33_ values, as studied by Yang et al. They obtained 21 pC/N with 90% β phase with 0.2 wt % MWCNTs addition.^38^ The rolling was operated at 50 °C, which dramatically increased the piezoelectric response of the PVDF film.

Among the composite PVDF films, as the β phase content increased, *d*_33_ values increased. However, it was assumed that other factors also affect the piezoelectric coefficient, which is an increment in dielectric strength with the addition of additives that can increase the polarizability of the polymer composite film or deterioration of the PVDF film due to the excess rolling process.

The lowest *d*_33_ was obtained by mBT+PVDF-R composites. This might be due to diminishing β phase crystalline structure by forming a barrier between PVDF and BT particles, which was shown in XRD and DSC analyses. Ye et al. also prepared modified BT containing PVDF polymer films and investigated the dielectric properties and energy density values of the PVDF film.^56^ Opposite to our findings, they obtained improvement in piezoelectric properties due to modifying the surface of BT particles with tetradecylphosphonic acid, which is more compatible with PVDF than GPTMS.

## 4 Conclusions

The effects of different processing methods, including solvent casting, hot pressing, and their combination with varying processing techniques such as stretching, quenching, rolling, and thermal treatment (i.e., plasma treatment and UV treatment), were studied with a corresponding analysis of their influence on PVDF phase content and thermal behavior. PVDF film composites, including MWCNTs and BT/mBT, were prepared by SC-HP-Q-R processes, and the effects of these additives on the phase and crystalline behavior of PVDF were investigated.

Stretching and rolling the samples increased the β phase content dramatically. Quenching following hot pressing was necessary to maintain the β phase content during cooling.

UV treatment was determined to increase the β phase content by acting as a photonic annealing step. However, plasma treatment did not improve the β phase content of solvent cast materials.

Adding MWCNTs and/or BT increased the crystalline β-phase structure of PVDF. The less positive impact of the addition of BT to the degree of crystallinity proved more dominant than the supposed improvements that should have been attained by adding MWCNTs in MWCNT+BT+PVDF composite films, as these materials exhibited slight increments in crystallinity. Modifying BT particles with a silane coupling agent aided with achieving a better distribution of the particles in the PVDF matrix, resulting in a relatively lower crystallinity and β phase content due to insufficient compatibility between PVDF and the coupling agent. This created barriers between the polymer and particle interface, leading to reduced piezoelectric response properties.

Samples processed by solvent casting possessed a porous structure that inhibited the polarizability of the material. Processing the solvent cast samples using hot pressing and stretching allowed for applying a high voltage during poling, thereby circumventing the problem. The piezoelectric effect was successfully attained in the material. A piezoelectric coefficient of 9.2 pC/N was subsequently measured for stretched PVDF, and a maximum of 4.7 pC/N was obtained for a rolled polymer composite containing MWCNTs. The study produced a high β phase PVDF film that was both flexible and polarizable.

Particle addition did not affect the glass transition or relaxation temperatures. However, incorporating MWCNTs improved elastic modulus due to particle dispersion and the high aspect ratio of MWCNTs.

## Supporting Information Available

Figure S1. Additional experimental results (FTIR) for solvent cast PVDF materials processed using different methods. Figure S2. Additional experimental results (XRD) for solvent cast PVDF materials processed using different methods. Table S1. Plasma treatment parameters. Table S2. DSC test results including % crystallinity and melting temperature of PVDF materials processed by different methods and polymer composites (PDF)
