# Supplementary material for: β Phase Optimization of Solvent Cast PVDF as a Function of the Processing Method and Additive Content
Source: ACS Omega. 2024 Jun 3;9(24):26020–9. doi: 10.1021/acsomega.4c01221 (PMC11190934; doi:10.1021/acsomega.4c01221)
Supplement: Supplementary file 1 — ao4c01221_si_001.pdf [file ao4c01221_si_001.pdf]

β phase optimisation of solvent cast PVDF as a function of the processing method and additive content

Miray Yasar[a], Patrick Hassett[a], Neal Murphy[a*], Alojz Ivankovic[a]

[a]School of Mechanical and Materials Engineering, University College Dublin, Dublin, Ireland

Corresponding author:

Neal Murphy

E-mail address: neal.murphy@ucd.ie

Postal address: University College Dublin, School of Mechanical and Materials Engineering, Engineering Building Belfield Dublin 4

Author Contributions

M.Y.: Conceptualization, Investigation, Methodology, Data analyses, Validation, Visualization, Writing – original draft, Writing – review & editing. P.H.: Investigation, Writing – original draft. N.M.: Supervision, Writing - review & editing. A.I.: Supervision, Funding acquisition, Project administration

ORCID iDs

Miray Yasar	https://orcid.org/0000-0002-6078-4201
Patrick Hassett	https://orcid.org/0009-0001-3510-2934
Neal Murphy	https://orcid.org/0000-0002-7236-0932
Alojz Ivankovic	https://orcid.org/0000-0002-3938-828X

Supporting Information

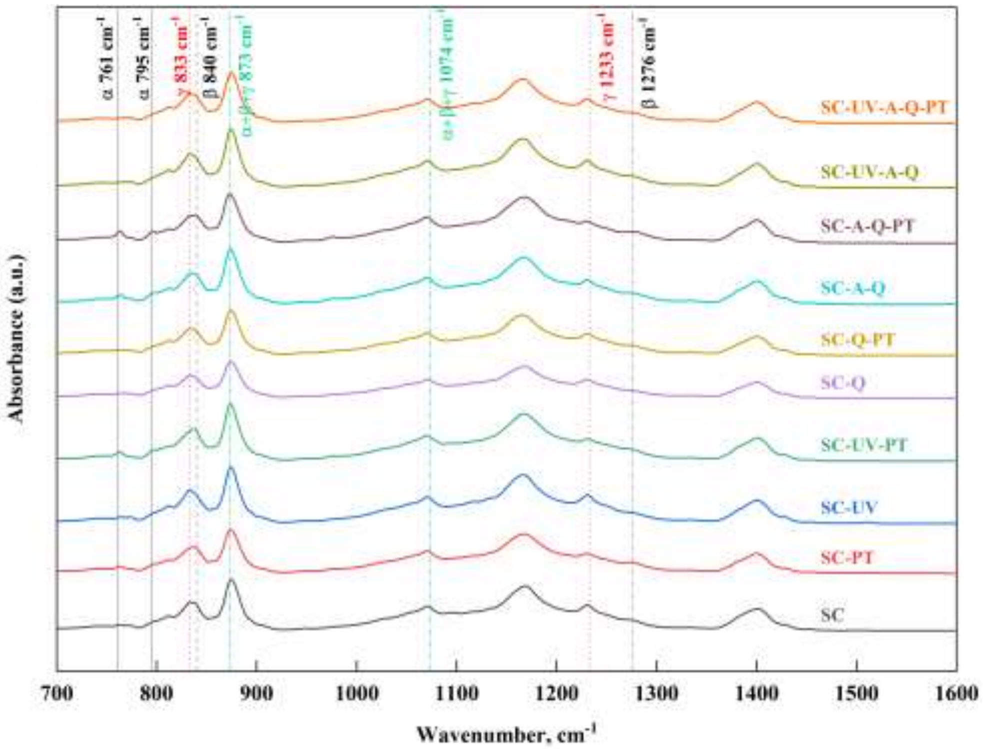

Figure S1. FTIR analysis of solvent cast PVDF materials processed using different methods.

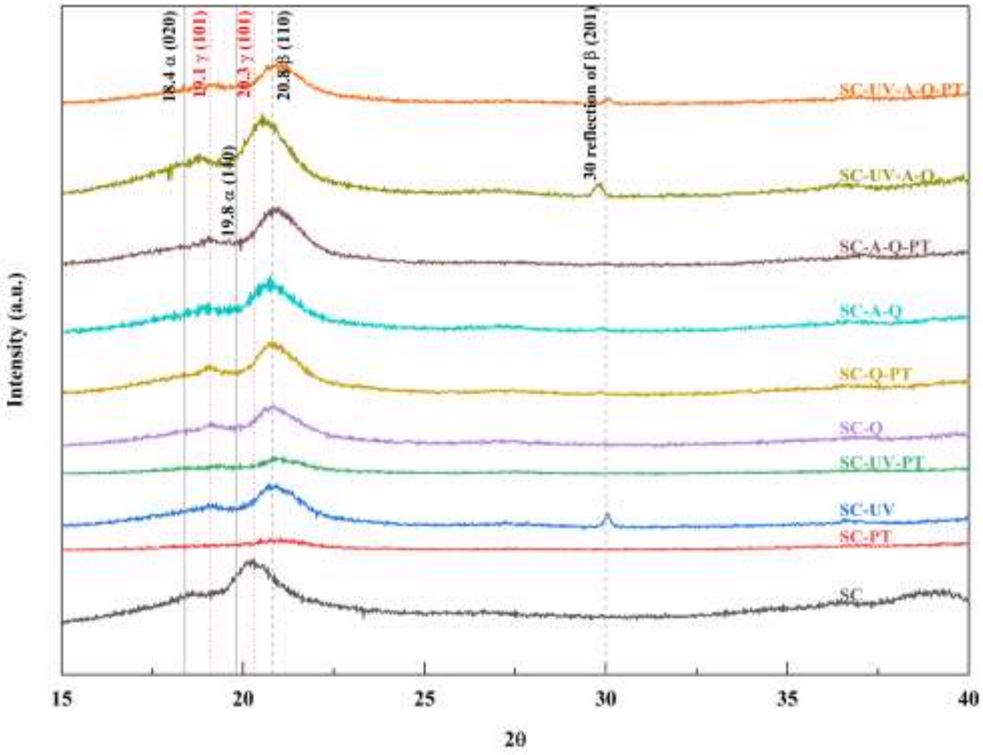

Figure S2. XRD analysis of solvent cast PVDF materials processed using different methods.

Table S1. Plasma treatment parameters.

**Plasma Parameters**	**Value**
PCT %	50
Voltage %	80
Frequency (kHz)	20
Ion. Gas Press. (l/h)	2400
Passes (#)	1
Z Height (mm)	15
Raster Spacing (mm)	1.5
XY Treatment Speed (mm/s)	200

Table S2. % Crystallinity and melting temperature of PVDF films processed by different methods and polymer composite films.

**Main Processes**	**Material**	**ΔH_f, J/g**	**X_c, %**	**T_m, ^oC**
**Solvent Cast PVDF**	SC	52.86	50.49	161.5
	SC-PT	45.18	43.15	168.5
	SC-UV	47.49	45.36	169.6
	SC-UV-PT	53.76	51.35	164.5
	SC-Q	48.06	45.90	168.5
	SC-Q-PT	47.99	45.83	169.4
	SC-A-Q	45.54	43.50	169.5
	SC-A-Q-PT	44.64	42.64	169.6
	SC-UV-A-Q	41.62	39.75	169.5
	SC-UV-A-Q-PT	41.88	40.00	169.5
	SC-HP-Q	39.04	37.29	162.5
	SC-HP-Q-S	41.10	39.26	159.5
	SC-HP-Q-R	42.24	40.34	159.6
**PVDF Composites (SC-HP-Q-R)**	BT+PVDF-R	45.42	43.38	159.5
	mBT+PVDF-R	42.74	40.82	157.6
	MWCNT+PVDF-R	46.56	44.47	160.5
	MWCNT+BT+PVDF-R	43.02	41.09	159.5
